# Targeting Bone Alleviates Osteoarthritis in Osteopenic Mice and Modulates Cartilage Catabolism

**DOI:** 10.1371/journal.pone.0033543

**Published:** 2012-03-14

**Authors:** Thomas Funck-Brentano, Hilène Lin, Eric Hay, Marie-Dominique Ah Kioon, Corinne Schiltz, Didier Hannouche, Rémy Nizard, Frédéric Lioté, Philippe Orcel, Marie-Christine de Vernejoul, Martine Esther Cohen-Solal

**Affiliations:** 1 INSERM U606, Centre Viggo Petersen and Université Paris-Diderot Paris 7, Hôpital Lariboisière, Paris, France; 2 Department of Orthopedics, Centre Viggo Petersen and Université Paris-Diderot Paris 7, Hôpital Lariboisière, Paris, France; 3 Department of Rheumatology, Centre Viggo Petersen and Université Paris-Diderot Paris 7, Hôpital Lariboisière, Paris, France; Institut de Génomique Fonctionnelle de Lyon, France

## Abstract

**Objective:**

Subchondral bone modifications occur early in the development of osteoarthritis (OA). The level of bone resorption might impact cartilage remodeling. We therefore assessed the in vivo and in vitro effects of targeting bone resorption in OA and cartilage metabolism.

**Methods:**

OA was induced by meniscectomy (MNX) in ovariectomized osteopenic mice (OP) treated with estradiol (E2), pamidronate (PAM), or phosphate buffered saline (PBS) for 6 weeks. We assessed the subchondral bone and cartilage structure and the expression of cartilage matrix proteases. To assess the involvement of bone soluble factors in cartilage metabolism, supernatant of human bone explants pre-treated with E2 or PAM were transferred to cartilage explants to assess proteoglycan release and aggrecan cleavage. OPG/RANKL mRNA expression was assessed in bone explants by real-time quantitative PCR. The role of osteoprotegerin (OPG) in the bone-cartilage crosstalk was tested using an OPG neutralizing antibody.

**Results:**

Bone mineral density of OP mice and osteoclast number were restored by E2 and PAM (p<0.05). In OP mice, E2 and PAM decreased ADAMTS-4 and -5 expression, while only PAM markedly reduced OA compared to PBS (2.0±0.63 vs 5.2±0.95; p<0.05). OPG/RANKL mRNA was increased in human bone explants treated with both drugs (2.2–3.7-fold). Moreover, supernatants from bone explants cultured with E2 or PAM reduced aggrecan cleavage and cartilage proteoglycan release (73±8.0% and 80±22% of control, respectively, p<0.05). This effect was reversed with osteoprotegerin blockade.

**Conclusion:**

The inhibition of bone resorption by pamidronate in osteopenic mice alleviates the histological OA score with a reduction in the expression of aggrecanases. Bone soluble factors, such as osteoprotegerin, impact the cartilage response to catabolic factors. This study further highlights the importance of subchondral bone in the regulation of joint cartilage damage in OA.

## Introduction

Osteoarthritis (OA) is a common disease responsible for high morbidity related to functional disability and pain. Although cartilage lesions are the main feature of OA, other joint structures also contribute to progression of the disease via several mechanisms. Radin *et al.* [1], [2] was the first to suggest that subchondral bone could trigger degradation of the overlying cartilage. Subsequently, studies on humans and animal models have revealed concomitant changes in bone and cartilage structures, particularly during the late stages of OA. In patients with established OA, trabecular bone volume is high, and bone accretion is correlated with the severity of cartilage damage [3]. Subchondral bone lesions appear during the early stages of OA, as demonstrated by the presence of bone marrow lesions that are factors predictive of OA progression [4], [5]. The natures of such bone lesions are still poorly established, although trabecular fractures or enhanced trabecular bone remodeling are suspected. Early stages of OA are accompanied by a decrease in bone volume, which suggests that initial bone resorption might trigger OA [6]–[9]. Indeed, OA can be prevented in rats by inhibiting bone resorption with alendronate [6]. Several molecules may mediate osteoclastic bone resorption, and thus have an impact on cartilage remodeling [10]. We and others have demonstrated that osteoprotegerin (OPG), a decoy receptor of Receptor Activator for Nuclear Factor κB Ligand (RANKL) produced by osteoblasts, could decrease joint cartilage degradation in a murine model of joint instability [10], [11]. Reducing bone remodeling in Runx2 transgenic mice prevents from cartilage breakdown [12].

The role of bone resorption related to estrogen failure in the development of OA is still a matter of debate. In humans, estrogen deficiency is reported to be a risk factor for OA, and estrogen deficiency/polymorphism appears to increase the incidence of OA in postmenopausal women [13]–[16], although this remains controversial [17]. In mice, the deletion of estrogen receptors does not affect the cartilage structure [18], whereas protective effects of estradiol in the development of OA have been shown in other animal models [19], [20]. Estrogens are powerful osteoclast inhibitors [21], and might contribute to OA protection via their effects in bone.

The aim of this study was to assess whether cartilage protection could be achieved by inhibiting bone remodeling in mice with high bone resorption induced by estrogen deficiency. We therefore investigated the impact of reducing bone resorption in osteopenic mice (OP) with joint instability, and the effects of bone-secreted cytokines on cartilage catabolism.

## Results

### Inhibitors of bone resorption restore subchondral bone architecture in OP mice

The bodyweight of mice remained stable in each group throughout the study (Table 1). Uterus weight declined after ovariectomy in the Phosphate Buffered Saline (PBS)-treated and Pamidronate (PAM)-treated mice, but was maintained in the mice treated with estradiol. Total body BMD decreased significantly in OP mice (p<0.05), and this was reversed by both pamidronate and estradiol. These results indicate an appropriate systemic response to both anti-resorptive therapies. BMD changes in the knee were in line with those of total body BMD.

**Table 1:** Effect of bone resorption inhibitors on bone indices in osteopenic mice with joint instability.

	Controls	Osteopenic mice		
		PBS	E2	PAM
Body weight (g)	22.1±0.52	22.4±0.64	23.9±0.50	21.4±0.42
Uterus weight (mg)	47±3	25±4§	68±6*	35±15
ΔBMD Total body (g/cm^2^)	0.039±0.006	−0.027±0.023§	0.124±0.014§ *	0.119±0.0 18§ *
ΔBMD Left knee (g/cm^2^)	0.053±0.024	−0.065±0.035§	0.178±0.048§ *	0.189±0.010§ *
ΔBMD Right knee (g/cm^2^)	0.141±0.023	−0.063±0.048§	0.216±0.043§ *	0.231±0.028§ *

§ : compared to controls, p<0.05.

* : compared to PBS, p<0.05.

Bone indices were measured at the tibia 6 weeks after MNX. Bone volume (BV/TV) was lower in OP mice than in controls, which was consistent with the increase in Tb.Sp and osteoclast number, and the lower Tb.Th (Table 2). Estradiol prevented the local bone loss observed in the OP-PBS mice (48.8±4.8% vs 27.6±3%, p = 0.005), and also prevented the increase in trabecular spacing and osteoclast number. The effects of estrogens on the local microarchitecture were in line with their effects on systemic bone. In contrast, pamidronate-treated mice had significantly lower osteoclast numbers, but no significant changes were observed in BV/TV (30.7±4.6% vs 27.6±3%, p = 0.48), although BMD data indicated that this treatment did prevent systemic bone loss.

**Table 2:** Histomorphometric parameters in the meniscectomized knees.

	Controls	Osteopenic mice		
		PBS	E2	PAM
BV/TV (%)	42±2.8	27.6±3§	48.8±4.8*	30.7±4.6
Tb.Th (µm)	12.5±0.98	9.8±0.53§	11.4±1.03	10.03±0.88
Tb.Sp (µm)	17.5±13.6	28.6±3.4§	12.6±1.7*	24.5±4.8
Tb.N (/µm)	0.034±0.002	0.028±0.003	0.043±0.004*	0.030±0.004
Oc.N/BV (/mm^2^)	17.4±6.3	45.7±10.5§	21.4±10.6*	19.9±7.9*

§ : compared to controls, p<0.05.

* : compared to PBS, p<0.05.

### Anti-resorptive agents protected mice against OA

**Figure 1:**
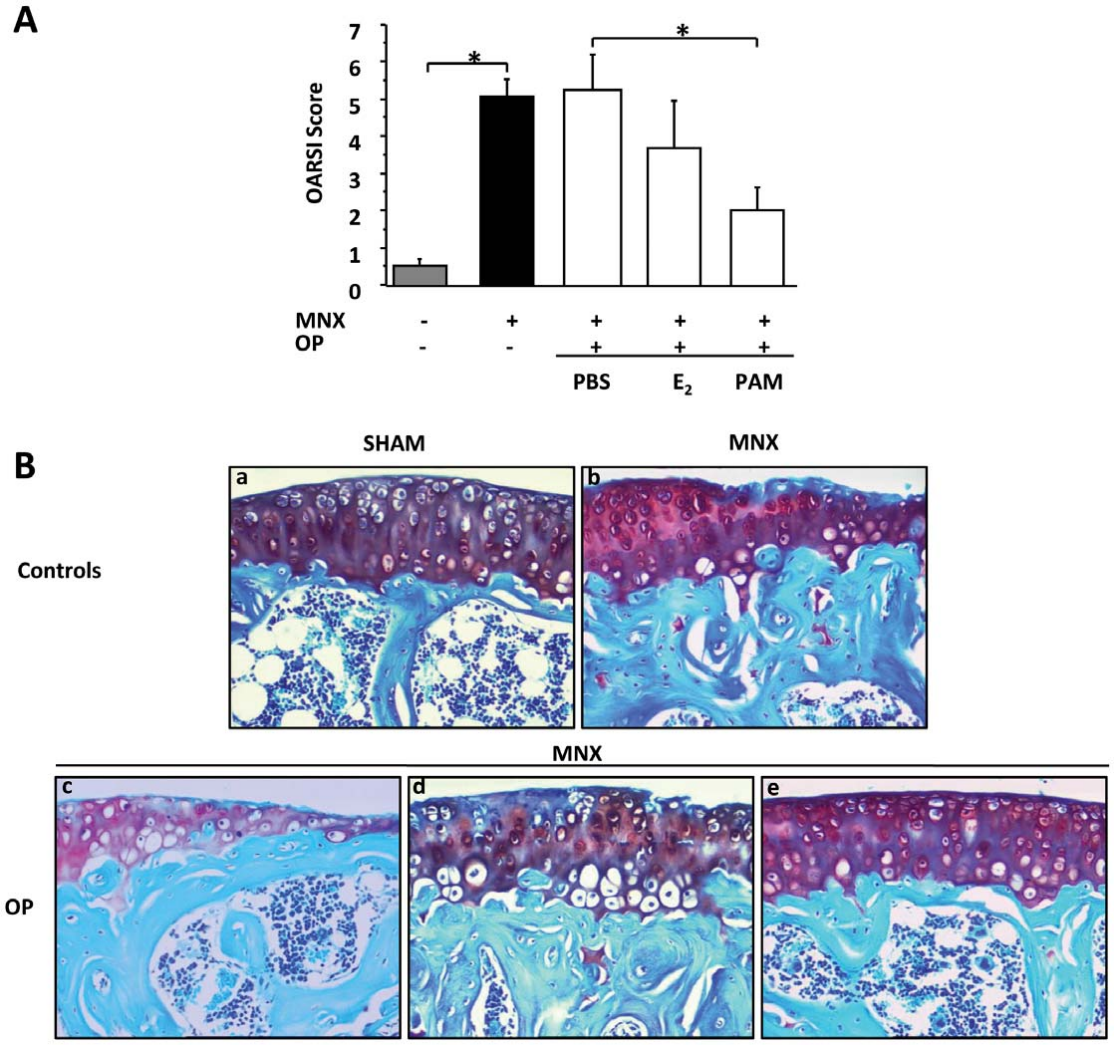
Histological assessment of cartilage lesions. A. OA scored according to the 2010 OARSI recommendations. The score represents the sum of the grades at the tibia and femur in sagittal sections. B. Magnifications of slides stained with safranin-O (40×) in control mice (a: Sham operated knees; b: MNX knees; n = 8) and osteopenic (OP) mice treated with phosphate buffered saline (PBS) (c; n = 10), estradiol (E_2_) (d; n = 6), or pamidronate (PAM) (e; n = 5). *: p<0.05.

The histological OA score was significantly greater after MNX than in the sham-operated knees (5.1±0.47 vs 0.5±0.18, respectively; p = 0.018) (Figure 1). However, OP did not produce any further increase in the OA score compared to controls (5.2±0.95; p = 0.86). In MNX knees, pamidronate provided significant reduction of cartilage degradation compared to PBS treatment (OARSI 2010 score: 2.0±0.63 vs 5.2±0.95; p = 0.042). In contrast, the effect of estradiol was not significant, although a similar trend was observed (3.7±1.30; p = 0.18) (Figure 1). These results show that pamidronate reduced OA in a context of high systemic bone resorption whereas estradiol deficiency or supplementation had no significant structural effects on cartilage degradation induced by MNX.

**Figure 2:**
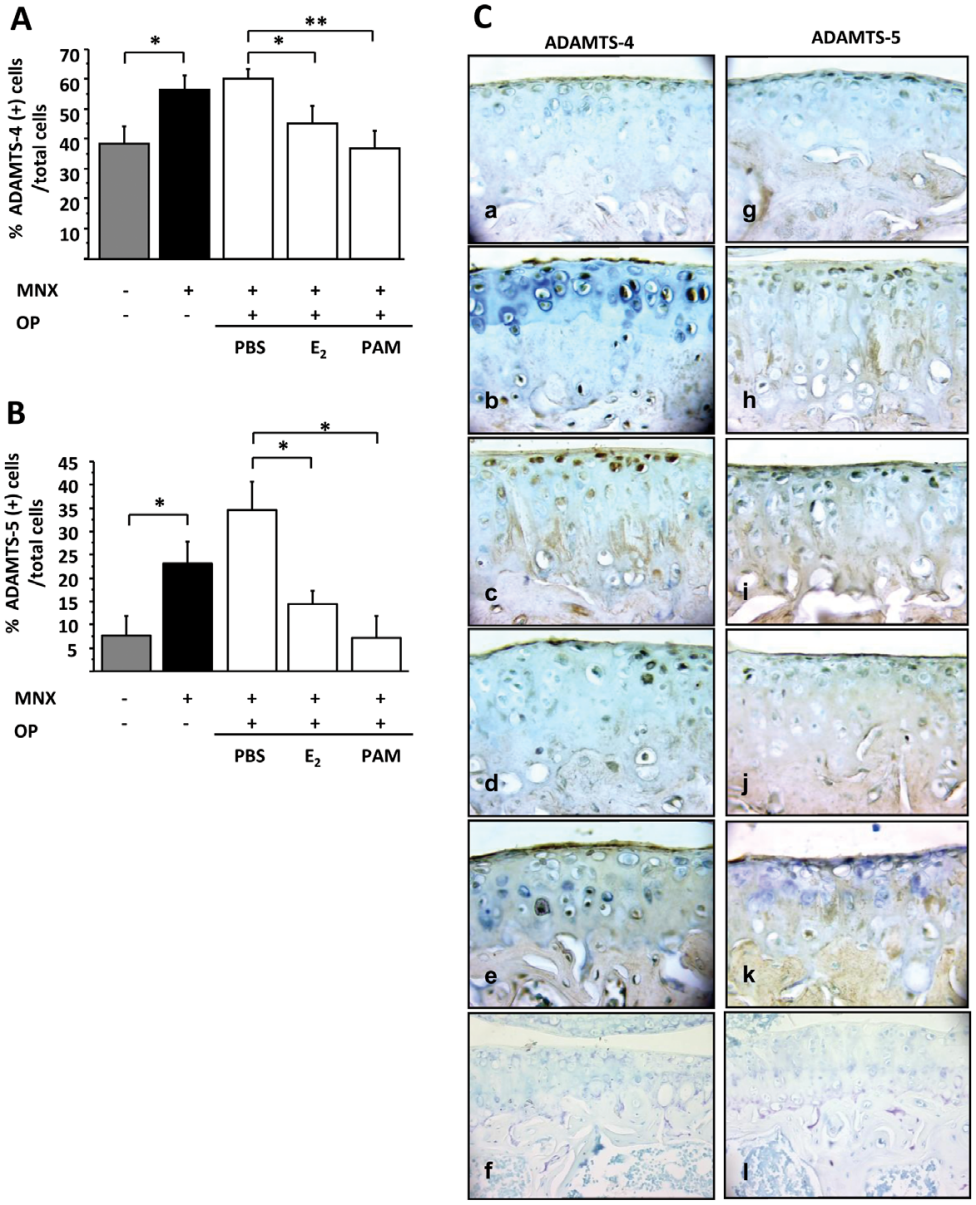
Expression of ADAMTS-4 and ADAMTS-5 in joint cartilage. A. Immunostaining for ADAMTS-4 in cartilage. Bars represent the percentage of positive cells. B. Immunostaining for ADAMTS-5 in cartilage. C. 40× magnification of ADAMTS-4 & ADAMTS-5 expression in the tibial joint cartilage in Sham operated knees (a–g), and MNX knees (b–h) of control mice, and in MNX knees from OP mice treated with phosphate buffered saline (PBS) (c–i), estradiol (E_2_) (d–j) or pamidronate (PAM) (e–k), with corresponding negative controls (f–l). *: p<0.05. **: p<0.01.

To further investigate the biochemical changes in cartilage in each group, we analyzed the expression of ADAMTS by immunohistochemistry (Figure 2). MNX increased the expression of ADAMTS-4 and ADAMTS-5 in chondrocytes compared to controls (positive cells for ADAMTS-4 and -5: 58.1±3.5% vs 38.8±5.8%; p = 0.017; 22.9±4.7% vs 7.5±4.3%; p = 0.027, respectively). The number of ADAMTS-4 expressing cells was lower after estradiol and pamidronate than in PBS-treated mice (45.2±5.9%, and 37.0±5.6%, vs 60.2±3.0%, p = 0.025 and p = 0.01 respectively). Similar results were observed with ADAMTS-5 (14.2±2.9% and 7.10±4.7% vs 34.5±6.1%, p = 0.025 and p = 0.039, respectively, Figure 2).

Thus, estradiol and pamidronate both reduced aggrecanase expression, whereas only pamidronate significantly decreased the OA score induced by MNX.

### Bone supernatants reduced human cartilage explants breakdown

**Figure 3:**
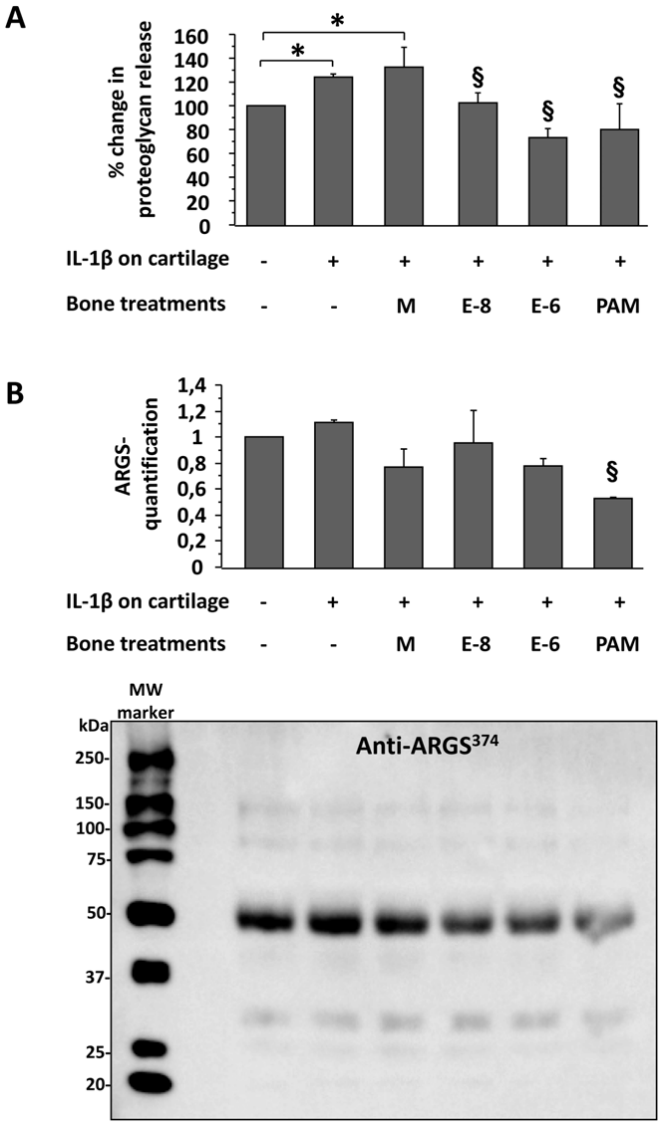
Bone mediated effects of estradiol and pamidronate on proteoglycan and aggrecan neoepitopes release by human cartilage explants. A. Proteoglycan release in cartilage supernatants. IL1-β stimulated cartilage explants were cultured with media from bone explants previously cultured with estradiol at 0.01 µM (E-8) or 1 µM (E-6) or pamidronate at 1 µM (PAM) or with control medium (M). Results are the mean ± SEM of 3 different experiments performed in triplicate. B. Aggrecan neoepitopes expression (ARGS^374^ sequences) in cartilage supernatants. Image of a representative Western blot. The overlying graph represents the mean quantification ± SEM of 3 different experiments. Quantification was done on the 50 kDa bands. *: compared to control, p<0.05. §: compared to IL-1β stimulation of cartilage, p<0.05.

To further understand the crosstalk between bone and cartilage, we investigated whether the conditioned media of bone explants contributed to the cartilage breakdown. IL-1β alone or in addition to the supernatants of control bone explants induced a significant increase in proteoglycan release from cartilage explants (123.9±2.4% and 132±16% of controls, respectively, p<0.05) (Figure 3). Moreover, culture supernatant from bone pre-treated with estradiol at 0.01 µM or 1 µM, or with pamidronate at 1 µM, was able to reverse cartilage breakdown (102±8.0%, 73±8.0% and 80±22% of control, respectively, p<0.05) (Figure 3A). The expression of aggrecan neoepitopes (ARGS^374^ sequences) was high in baseline conditions, the cartilage explants being harvested from patients with severe OA. However, this level was reduced by adding the supernatant of bone cultured with pamidronate, but not with the 2 doses of estradiol (Figure 3B).

### OPG contributes to the bone-cartilage crosstalk

**Figure 4:**
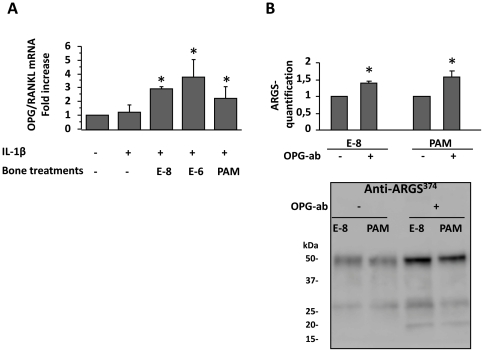
OPG exerts protective effects on cartilage catabolism. **A.** OPG/RANKL mRNA ratio in IL1-β stimulated bone explants cultured with estradiol at 0.01 µM (E-8) and 1 µM (E-6), and with pamidronate (PAM). The graph represents the mRNA ratio to controls in 3 different experiments. **B.** Effect of OPG blockade by a neutralizing antibody (OPG-ab) on ARGS^374^ sequences expression in supernatants of cartilage explants cultured with conditioned bone media. Image of a representative Western blot, and the graph shows the mean quantifications ± SEM of 4 different experiments. Quantification was done on the 50 kDa bands. *: p<0.05.

To find out whether cytokines were involved in this effect, the expressions of RANKL and OPG transcripts were measured. Adding IL-1β did not increase the OPG/RANKL mRNA ratio in bone explants (1.2±0.55 times control, p = 0.43, Figure 4A). Bone cultured with E2 showed a significant, dose-dependent increase in the OPG/RANKL ratio (2.9±0.16 and 3.7±1.3 times control, at 0.01 and 1 µM, respectively, p = 0.019), as did those cultured with PAM (2.2±0.9 times control, p = 0.028). To find out whether the effect of conditioned bone cultures was mediated by OPG, OPG neutralizing antibody was added to the bone culture medium before it was transferred onto cartilage explants. We observed that OPG neutralization reversed the protective effects of E2 and PAM on aggrecanase neoepitopes expression (Figure 4B).

## Discussion

In this study, we show that regulating the level of bone remodeling in OP mice modulates the response of cartilage to meniscetomy. The inhibition of high bone resorption, here induced by ovariectomy, reduced cartilage lesions and the increase in both ADAMTS-4 and ADAMTS-5, the main aggrecanases involved in OA [22], [23]. These results are consistent with our previous observations showing that pamidronate reduced cartilage damage in Runx-2 transgenic mice with high bone resorption, but had no effect in control littermates with normal bone remodeling [12]. Altogether, these results show that pamidronate was effective only in mice with high resorption, suggesting that its action is driven by bone. Thus, in the murin model of OA by joint instability, the inhibition of high osteoclast activity has some protective effects on the overlying cartilage.

At 6 weeks after MNX, ovariectomy induced no additional damage, in contrast with previous observations in rats or rabbits [24], [25]. In our study, the effect of ovariectomy could have been minimized by the local effect of MNX. Mice were treated with estrogens or with pamidronate to target osteoclast formation. Along with the reduction of osteoclast number, pamidronate reduces cartilage structural lesions both at the tissue and cellular level. Estrogens, that inhibit osteoclasts, failed to provide significant protection against cartilage structural lesions, despite reducing ADAMTS expression. However, subchondral bone was restored with estrogens along with the prevention of systemic bone loss. These differences suggest that these drugs may have differential impacts on subchondral and trabecular bone in OP mice. These results demonstrate the role of subchondral bone, and in particular of osteoclast activity, in the initiation of OA lesions.

Despite clinical evidence showing that OA is more frequent in women, the contribution of estrogen in the pathogenesis of OA remains unclear and might be mediated by the effects of bone resorption. Estradiol modulates the chondrocyte phenotype *in vitro* via both the ERα and ERβ receptors [26]–[29]. *In vivo*, studies have shown that ovariectomy has harmful effects on joint cartilage [8], but conflicting findings have also been reported [19], [30]. Osteoporosis induced in rabbits using both ovariectomy and corticosteroids experienced cartilage damage [8] further indicating that cartilage lesions might be induced by bone signals in addition to a direct effect on chondrocytes.

To investigate the mechanisms by which bone cell activity could modulate cartilage metabolism, we transferred supernatant of human subchondral bone cultures on cartilage explants. Indeed, we found that the release of proteoglycan and aggrecan neoepitopes in cartilage cultures was reduced by bone supernatants cultured with estradiol and pamidronate, indicating that bone-secreted soluble factors are involved in regulating cartilage catabolism. Since chondrocytes express RANK and RANKL [31]–[34] and treatment with OPG showed protective effects in the joint instability model [10], [11], we speculated that OPG could mediate the effect of estradiol or pamidronate. Hence, we found that the OPG/RANKL ratio was significantly higher in bone explants cultured in the presence of estradiol and pamidronate. The inhibition of OPG in the bone culture medium transferred into cartilage cultures resulted in a marked increase in aggrecanase activity, demonstrating the role of OPG in the regulation of cartilage catabolism. Indeed, OPG/RANKL ratio is reduced in subchondral bone of OA rabbits with osteoporosis [8] as well as in rat mandibular condyles [33]. The effect of OPG can be mediated by its interaction with TRAIL, and thus via regulation of chondrocyte apoptosis, as suggested by Shimizu et al. [11]. Taken together, these data indicate that OPG and RANKL are involved in a two-way crosstalk between bone and joint cartilage, regulating both cartilage catabolism and subchondral bone resorption. How these tissues can communicate has long been a subject of debate given the absence of vascularization in cartilage, and the high mineralization of calcified cartilage. In addition to the neo-vascularization of subchondral bone, the diffusion of local factors through bone clefts or through canaliculae in calcified cartilage seems likely to occur [35].

In conclusion, our study shows that bone is implicated in the mechanisms leading to OA, and that the level of bone remodeling modulates how cartilage responds to joint instability. Moreover, OPG is an essential factor that regulates cartilage catabolism. This study further highlights bone as a target for the treatment of OA.

## Methods

### Animals

Ten week-old female C57/Bl6 mice underwent medial meniscectomy (MNX) of the right knee to induce joint instability, as previously described [10]. A sham operation was performed on the left knee. To increase bone resorption, we induced osteopenia (OP) by ovariectomy and treated mice by two different bone resorption inhibitors: estradiol and a bisphosphonate (pamidronate) to inhibit osteoclast activity. All ovariectomies were performed in a random order regardless of the groups. After meniscectomy, mice were divided into 4 groups:

3 groups of 5–10 animals underwent bilateral ovariectomy to induce OP. These mice received either subcutaneous daily injections of 17β estradiol (15 µg/kg/d, OP-E2), or intra-peritoneal injections of pamidronate (30 mg/kg/w, OP-PAM), or Phosphate buffered saline (100 µl, OP-PBS).
1 group was sham operated without ovariectomy, and was used as the control group.

Mice were pair-fed in order to control for bodyweight, and were sacrificed 6 weeks after surgery. The uterus was removed and weighed.

Whole-body bone mineral density was measured using a GE Lunar Piximus (GE Healthcare, WI, USA) at baseline and at sacrifice (BMD, g/cm^2^) in order to assess whether the doses of estradiol and bisphosphonate were appropriate. The relative change in BMD from baseline to sacrifice (ΔBMD) was calculated as (BMD at sacrifice - BMD at baseline)/BMD at baseline.

The experiments complied with the Guidelines for Animal Experimentation issued by the local Ethics Committee on Animal Care and Experimentation (Ethical committee Lariboisière-Villemin, Paris, France). This committee specifically approved this study.

### Preparation of mouse joint samples

Whole knee joints were dissected free of soft tissues. Specimens were fixed with 4% PFA (pH 7.4) for 24 h at 4°C, and then decalcified with 1% PFA-0.2 M EDTA (pH 7.4, 4°C) for 2 weeks, changing the solution twice a week. The specimens were then dehydrated using increasing concentrations of ethanol, before being embedded in paraffin. Five-µm thick serial sagittal sections were cut in the medial femoro-tibial compartment for histology and immunohistochemistry procedures.

### Histology and immunohistochemistry

We assessed the histological OA score on slides stained with safranin-O. Sections were deparaffinized by two successive 20-minute immersions in xylene baths. They were then rehydrated by immersion for 1 min in each of 3 successive baths containing 100%, 70%, and 40% alcohol, respectively, before being washed twice by 5-min immersions in baths of distilled water. Sections were stained using Mayer-Hemalum staining for 5 min to stain the nuclei, and then counterstained with 0.125% Fast Green for 2 minutes to visualize bone tissue. After being rinsed in two successive 1% acetic acid baths, they were stained in 0.5% Safranin-O in the same bath, and then rinsed in 95% ethanol. All stained sections were then rinsed with distilled water. Osteoarthritic lesions were determined using the OARSI 2010 score with a scale ranging from stage 0 (normal) to 6 (vertical clefts/erosion to the calcified cartilage extending over >75% of the joint surface) on both the tibial and femoral articular cartilage, resulting in a global score of 0 to 12 [36].

Immunohistochemistry was performed on serial sections, as previously described [10]. Vector kit (PK-6101, from Abcys, France) was used according to the Manufacturer's instructions, and sections were counterstained with toluidine blue. Primary polyclonal antibodies directed against murine ADAMTS-4 and ADAMTS-5 (ab28285 and ab41037, respectively, Abcam, United-Kingdom) were used to assess aggrecanase expression and activity. Positive cells were counted on the tibial joint cartilage surface (25× magnification), and expressed as a percentage of the total cell count.

### Histomorphometry of subchondral bone

Bone parameters were measured on slides stained with Fast-Green. Microarchitectural indices of the underlying bone were assessed in total epiphyses by an image analyzer using specially designed software (Bonolab, Microvision®, France). For each section, the bone between the cartilage and the growth plate was analyzed, and measured as follows:

Bone Volume/Tissue Volume (BV/TV, %)
Trabecular Thickness (Tb.Th, µm), which reflects bone formation
Trabecular Separation (Tb.Sp, µm), which reflects bone resorption.
Osteoclast number (N. Oc/BV, /mm^2^)

### Human explant cultures

Human cartilage samples were harvested from patients who were undergoing total knee replacement surgery for medial OA, in accordance with the French National Authority Legislation for the collection of human tissues. This study was specifically approved by the Institutional Review Board: IRB N° 0000383). As required by the French bioethics law and the local IRB, the need for informed consent was waived since these tissues were surgical waste of routine joint replacement surgery, and since there was no patient private information being collected. All participants received an information note explaining the purpose of the study and were asked for their non opposition to participate.

Samples were collected from the posterior surface of the unaffected femoral condyle, using a bone-marrow trephine that ensured that the explants removed were equal in size (3.5-mm diameter). Bone and cartilage explants were separated mechanically, and cultured separately. All cultures were conducted in a humidified atmosphere of 95% air-5% CO2. After culturing for 72 h in BGJb medium (US Biological, MA, USA), bone explants were serum starved for 24 h hours, and the pre-stimulated with IL-1β at 10 ng/ml for 24 hours (R&D systems, France). They were then cultured for 48 h with 0.01 or 1 µM (E^−8^ or E^−6^) 17β-estradiol (Sigma-Aldrich, MO, USA), or 1 µM pamidronate (PAM). The explants were then washed and cultured in fresh medium for 24 hours. Cartilage explants were cultured in phenol-red free DMEM with 10% FCS and a cocktail of antibiotics until being subjected to stimulation with IL-1β at 10 ng/ml for 24 hours. Bone culture supernatants were then transferred onto cartilage explants for 72 h.

The proteoglycan content in the media was measured as sulfated glycosaminoglycan by a colorimetric assay using dimethylmethylene blue [37]. Aggrecan neoepitopes were analyzed by Western blotting using a monoclonal antibody that recognizes the human aggrecan neoepitopes ARGS^374^ sequences (ab3773, Abcam). The culture media (15 µl) used for each condition were deglycosylated with hyaluronidase at 0.3 mg/ml for 30 min, and then with chondroitinase ABC (Sigma-Aldrich) at 0.1 U/ml in a buffer containing 50 nM sodium acetate, 0.1 M Tris-HCl, pH 6.5, at 37°C for 90 min [38]. Samples were then analyzed by SDS-PAGE on 8% polyacrylamide gels following standard procedures.

Finally, to investigate the role of OPG released from bone in cartilage breakdown, OPG neutralizing antibody, OPG-ab (MAB805, R&D systems, France) or isotype control was added to the supernatant of bone cultures, and then transferred to cartilage explants for 72 hours. Supernatants were then tested for aggrecan neoepitopes by Western blotting.

### Bone mRNA extraction and gene expression

Human bone explants were then collected and processed for RNA extraction. Bone samples were ground in liquid nitrogen, and placed in 1 ml Trizol (Invitrogen, UK) for RNA extraction by centrifuging in chloroform and isopropanol, according to the manufacturing protocol. Total RNA were transcripted into cDNA by the cDNA verso Kit (Thermo Fisher Scientific, UK). Relative mRNA levels were evaluated by quantitative PCR analysis using LightCycler (Roche Applied Science, France) in Absolute Blue qPCR SYBR Green (Thermo Fisher Scientific), at a fusion temperature of 60°C, with 40 cycles and normalized to GAPDH mRNA using the following primers:

OPG-F: CTTTGGTCTCCTGCTAACTC

OPG-R: GAAGAATGCCTCCTCACACA

RANKL-F: GGCCTTTCAAGGAGCTGTGCAAAA

RANKL-R: AGCTTGCTCCTCTTGGCCAGATCTAA

GAPDH-F: ACAACAGCCTCAAGATCATCAG

GAPDH-R: CTGTGGTCATGAGTCCTTCCA

### Statistical analysis

All statistical analyzes were performed using STATVIEW® software (SAS Institute, NC, USA). Results are expressed as the mean ± SEM of at least 3 different experiments. The effects of meniscectomy and bone resorption inhibitors were analyzed using the Wilcoxon rank and Mann-Whitney tests. The latter test was also used to analyze the different conditions of culture of the human explants.
